# Demand segmentation and sociodemographic aspects of food festivals: A study in Bahrain

**DOI:** 10.1371/journal.pone.0287113

**Published:** 2023-06-14

**Authors:** Mauricio Carvache-Franco, Tahani Hassan, Orly Carvache-Franco, Wilmer Carvache-Franco, Olga Martin-Moreno

**Affiliations:** 1 Universidad Espíritu Santo, Samborondón, Ecuador; 2 Brunel Business School, Brunel University London, London, United Kingdom; 3 Facultad de Economía y Empresa, Universidad Católica de Santiago de Guayaquil, Guayaquil, Ecuador; 4 Facultad de Ciencias Sociales y Humanísticas, Escuela Superior Politécnica del Litoral, ESPOL, Guayaquil, Ecuador; Babes-Bolyai University, Cluj-Napoca, ROMANIA

## Abstract

Food festivals have been a growing tourism sector in recent years due to their contributions to a region’s economic, marketing, brand, and social growth. This study analyses the demand for the Bahrain food festival. The stated objectives were: i) To identify the motivational dimensions of the demand for the food festival, (ii) To determine the segments of the demand for the food festival, and (iii) To establish the relationship between the demand segments and socio-demographic aspects. The food festival investigated was the Bahrain Food Festival held in Bahrain, located on the east coast of the Persian Gulf. The sample consisted of 380 valid questionnaires and was taken using social networks from those attending the event. The statistical techniques used were factorial analysis and the K-means grouping method. The results show five motivational dimensions: Local food, Art, Entertainment, Socialization, and Escape and novelty. In addition, two segments were found; the first, Entertainment and novelties, is related to attendees who seek to enjoy the festive atmosphere and discover new restaurants. The second is Multiple motives, formed by attendees with several motivations simultaneously. This segment has the highest income and expenses, making it the most important group for developing plans and strategies. The results will contribute to the academic literature and the organizers of food festivals.

## 1. Introduction

Food festivals create tourist awareness of the region, improve the destination’s image, and show the region’s culture [1–3]. According to Fontefrancesco and Zocchi [4], food festivals are public events to celebrate specific food products with specific names that identify the events.

Visitor motivations are essential in developing a culinary image, area branding, and visitor loyalty to a destination [5]. In addition, studying visitors’ motivations allows for providing offers that fit their needs, tastes, motives, and demand [6]. The segmentation of tourists from a specific destination into clusters which address visitors’ relationship with food, is relevant because food is seen differently from one person to another [7, 8]. For example, some tourists consider food an essential element of their travel experience, while others think it is only necessary for energy during their trip [9]. Likewise, socio-demographic factors have an impact on tourists’ experience. Hence, since various visitors attend festivals, differences in age, nationality, marital status, daily expenses, and occupation may impact their experience, perception of food, attendance frequency, and participation in the festival’s activities. Therefore, it is crucial to investigate the motivations for attending food festivals and how they relate to socio-demographic factors [10–12].

Furthermore, in Bahrain, an Asian state located on the west coast of the Persian Gulf, the sixth version of the Bahrain Food Festival was held from March 19 to April 1, 2022 [13]. The Bahrain Tourism and Exhibition Authority, (BTEA) coordinated this festival. It was held in the Bahrain Harbour with over 100 local and international cafes offering t food varieties to suit all preferences [13]. The celebration incorporated a live cooking station, a children’s play region, musical performances by global and Bahraini artists, and contests in collaboration with lodges and restaurants such as Gulf Hotel [14]. Since it is a free event, the celebration aimed to promote Bahrain’s culinary variety and enhance the site as a tourism destination, especially after the prolonged COVID-19 lockdown (Bahrain New Agency BNA) [15, 16].

Researching Bahraini food celebrations is relevant for several reasons. First, scholars argued that food celebration studies are an underestimated research field. [17]. Second, as the information from scholars indicates, studies about the Bahrain Food Festival need to be conducted. Investigating this celebration could help the organizers to work more efficiently in the event, organize and execute exhibition methodologies to attract more guests, and improve the economy. The present study concerning the Bahrain Food Festival has the following objectives: (i) Identify the motivational dimensions of the demand for the food festival, (ii) Determine the segments of the demand for the food festival, and (iii) Establish the relationship between demand segments and sociodemographic aspects. The research question involves investigating the segments of the demand for motivations in the Bahrain food festival.

The results will serve as management guides for food festival managers to promote designs that support a proper rotation of events and for private companies to promote their products based on the interest found in their visitors. Likewise, it will contribute to the academic literature on festivals that still needs to be explored.

## 2. Literature review

### 2.1. Motivations to attend food festivals

Food festivals are considered one of the most popular events held worldwide. Food festivals are popular because they satisfy an essential human need, and provide visitors with an emotional and pleasurable experience [8]. In addition, food festivals may help promote the destination’s food culture and improve the economy [18]. Food festivals are public events that celebrate and promote specific food products, such as the Melbourne Food and Wine Festival, 2022; Maine Lobster Festival, 2022; and Bahrain Food Festival, 2022 [4]. Nowadays, gastronomy constitutes a significant motivation to undertake a trip. Thus, food provides a motivational draw for travel, such as visiting attractions, shopping for food products, having a dining experience, and learning about the destination’s culture [19, 20]. In this regard, Moutinho [21] states that motivation refers to the need or condition that drives people to act in a way that grants satisfaction.

Gastronomy is considered a fundamental factor that motivates tourists to visit a specific destination [22, 23]. Therefore, lacking motivation will diminish spending time and money on food and affect the destination’s image [7]. Previous findings have been made about motivations to attend food festivals. For example, Horng et al. [24] pointed out that the motivations to attend food festivals are to enjoy the event, taste the food, spend time with the family, and escape from the daily routine. Along the same lines, López-Guzmán et al. [22] identified the following motives: to enjoy a new food experience (taste food), culture (learn about the different cultures and flavors of local food), and socialization (increase family and friendship ties and exchange ideas about food with other visitors). Similarly, Kim et al. [25] determined five motivations for visiting food festivals: interpersonal relationship, enthusiasm, cultural experience, and taste satisfaction. Moreover, Hattingh and Swart [26] claimed that the reasons for visiting food festivals were to eat food, taste wines, enjoy new environments, see the live show of famous chefs, relax, and spend time with family. Also, Chang [27] studied The Old Town Spring Festival in Texas (USA) and identified similar motivations for attending, such as to taste wine, escape/event novelty, taste food and learn about cuisines, socialize, and enjoy art.

On the contrary, other studies divided the motivations to visit food festivals into reasons of pull and push [28, 29]. According to Yuan et al. [29], the pull motives include the services and the festival program, while the pull factors include socializing, enjoyment, and relaxation. Similarly, Smith et al. [30] added that push motives (individuals’ internal factors) encompassed the socialization and novelty of the festival. At the same time, pull factors (external factors related to the destination of the festival) comprised the provision of services and food products. It can be inferred that there is no set of motivations for all food festivals in the world due to the festival’s theme, location, and visitors’ socio-demographic characteristics [31].

A careful look at the studies on the motivations for visiting food festivals in different countries showed similarities, with a slight difference attributed to the nature of the festival, the program, and the activities of the event. For example, Topole et al. [32] studied five culinary events on the Slovenian karst plateau (Kras). They found that the reasons for visiting these events are to sample local cuisine, explore cultural heritage, and experience a new or different experience. The study of the Red Crab festival in Ecuador showed that visitors are motivated by six dimensions: escape, socialization, art, food, local food, and knowledge [33]. In a study on The Hefei, China, Crawfish Festival conducted by Hu et al. [34], the reasons for attending this event are classified into two sets: visitors’ motivations related to the event (novelty, culture, entertainment, relaxation, family bonding, socialization, escape, and excitement) and food-related motivations (physical environment, sensory appeal, prestige, celebration, social context, knowledge about food, and food culture). Another study on the Dalian International Wine and Dining Festival in China revealed that the motivations to attend were to enjoy the festival events, family unity, life balance, cultural exploration through socialization, and interaction with other visitors and participants [35]. Similarly, escaping from routine and enjoying time with family/friends were e reasons to visit the Urla International Artichoke Festival in Izmir, Turkey [36]. Differently, from the classification of the reasons for visiting food festivals, Kabiraj et al. [37] pointed out two sets of motivations for attending the Dalian International Wine and Dining Festival. Those are primary motivations to try and buy wine and food, experience wine and food culture, and participate in specific activities like wine and food tasting. Secondary motivations included being with family, meeting new friends with the same interests, and releasing pressures and changes in daily routine. In a recent study of the Wine and Food Festival in Miami, Florida (USA), three motivations for festival visitors were found: socializing with other visitors with identical interests, novelty seeking (such as enjoying variations of things to do and memorable experiences, festival program, and pleasure [38]. The following reasons were cited by Castillo-Canalejo et al. in [39] for visiting two food markets in Córdoba (Spain): Experience and novelty in food, hedonism, and leisure. Moreover, entertainment and socialization, loyalty, gastronomy, and novelty were identified as the elements that motivated visitors attendance in a research on a craft beer festival in Southern Africa [40].

In conclusion, the reasons for visiting food festivals vary due to the different characteristics of the event and the destination. However, common motifs exist among those festivals, such as socializing, novelty, relaxation, knowledge (learning), and food. Hence, the first research question in this study is.

RQ1: What are the motivations behind the visitor demand at the Bahrein food festival?

### 2.2. Segmentation of demand in food festivals

Demand segmentation refers to the market division into subgroups based on specific demographics, characteristics, lifestyles, and psychographic variables [8]. Classifying tourists according to segmentation demand and studying their characteristics and motivations can provide the necessary information to create products aligned with the needs and tastes of each tourist group [7, 41]. Hence, these products could improve tourist satisfaction and enhance their loyalty to the destination [7].

There are three perspectives to segment tourists concerning gastronomy. The first segmentation is based on the relationship between the visitors and the gastronomy affected by their visit motivations. The second is destination attribute-based segmentation which derives from the visitors’ decision to visit the destination and their relationship with its gastronomic attributes. Third, the economic-based segmentation is built on the economic impact of visitors to that destination [7]. Other studies provide a complete model for the segmentation of gastronomic tourists. Among them are the studies by Björk and Kauppinen-Räisänen [42] and López-Guzmán et al. [22] that classify tourists based on food and eating as travel reasons; the importance of the eating experience when selecting a destination and the relevance of eating for satisfaction during the trip. In addition, they indicated the existence of three groups of tourists’ *experimenters*-those who travel to gain experience in food; *enjoyers*-those who have positive attitudes toward food; *survivors*- those who have little or no interest in food. Some studies investigated the segmentation of festivalgoers, which segmented visitors considering different criteria. For example, Hsu et al. [8] highlighted that the Macau International Food Festival visitors were segmented according to three experiential value dimensions (including food and services, enjoyment and wellness, cost, and time factors). They classified visitors into four groups: Group 1- the low experiential value group: this cluster includes visitors who showed a low level of the three experiential value dimensions; Group 2—the moderate experiential value group: consists of visitors who showed moderate or neutral experiential values towards the food festival; Group 3—the multi-experiential value group: visitors in this cluster are those who showed high level towards all the experiential value dimensions towards the food festival; and, Group 4 food experiential value: this is the largest cluster, and it consists of those visitors who showed the highest level of food and service experiential value than other dimensions. According to motivations, Castillo-Canalejo et al. [39] divided the patrons of two Córdoba (Spain) food markets into three clusters. The first clusters are people who go to the market to try new foods and beverages, explore new activities, and spend time with family and friends. The second cluster consists of people who go to the market to socialize with family and friends, relax and disengage from daily life, and take advantage of its notoriety and reputation. The third cluster consists of people who travel to conduct business. Viljoen et al. [41] identified three groups of visitors to a South African food festival in a different study. Segment 1, referred to as "Social Epicureans," and drawn to the festival primarily as a kind of escape and socialization. Serious epicureans were categorized as segment 2’s visitors motivated by the entire experience.

Segment 3 was dubbed "Selective Epicureans" because these individuals valued specific elements of the experience, such as culinary exposure, and favoured qualities they particularly valued. These individuals chose escapism, socialization, product offerings, and uniqueness as their top motivators. Serious epicureans were categorized as segment 2’s visitors because they gave all five motivating aspects the highest ratings and appeared to be encouraged throughout the encounter.

Therefore, it is evident that food festival organizers face the challenge of offering different types of food and unforgettable and enjoyable experiences to the festival goers to maintain the event’s popularity. Thus, the segmentation of goers is vital to the festival’s success. In addition, segmentation simplifies the planning and formulation of strategies aligned to the specific needs of each group of food festival visitors [8]. Finally, agreement on the criteria is vital for segmenting festival visitors. Therefore, further research on the segmentation of food festivals is essential. This fact leads to the second research question:

RQ2: What are the segments of visitor demand in the Bahrein food festival?

### 2.3. Relation of the segmentation of the demand with the sociodemographic aspects in the food festivals

The sociodemographic aspects of the Valtice Wine Markets festival involve categorizing attendees based on their social and demographic characteristics, such as gender, age, education, income, marital status, and employment, to make them suitable for all visitor groups [23]. Generally, the studies to relate the sociodemographic aspects with other factors use these indicated variables. Lee et al. [43]. Previous research underscored the value of exploring the sociodemographic characteristics of guests at food festivals because it would help organizers provide better event services, infrastructure, programs, and sales. [11, 23, 44, 45].

Furthermore, previous findings in the literature showed the connection between the sociodemographic aspects of visitors to food festivals and the segmentation of demands. In the case of the Croatian Food and Wine Festival Feŝta in Adelaide, visitors were divided into two groups: Croatian-born visitors and non-Croatian-born visitors. Regarding visitor age group and education, Cluster 2 had the youngest visitors with a college education. In another study of festivals related to work and income, no significant differences were found between the two groups of visitors [46]. In a different study, Kim et al. [17] identified three groups of visitors to a food festival held in Oxford, Mississippi: Apathetic attendees: they visited the festival for "business or task,” satisfied spenders those visitors with a high level of perceived value satisfaction, and tentative tag-a-longs are visitors with a medium level of satisfaction and perceived value. Regarding gender, satisfied spenders had the highest number of males and females between 50 and 6Concerning to age, satisfied spenders had both the youngest and oldest groups of visitors. Regarding income, Apathetic attendees had the lowest income level compared to satisfied spenders and tentative tag-a-longs. Concerning education, satisfied spenders had the highest level of educated visitors, especially Bachelor’s and Graduated degrees. Finally, satisfied spenders had the highest level of single and married visitors compared to Apathetic attendees and tentative tag-a-longs. From a different perspective, Saayman et al. [47] divided visitors to the Wacky Wine Festival in South Africa into two groups based on their spending: the low-spending group and the high-spending group. Academics found a significant difference between these groups, which are based on sociodemographic aspects. The results showed that women spend more than men. Moreover, older visitors spend more than younger visitors because they consume more wine than the former. As for occupation, the high-spending cluster has more visitors from high-income professions. Thus, the third research question in food festivals arises:

RQ3: What is the relationship between the visitor demand segments and their sociodemographic variables in the Bahrein food festival?

## 3. Bahrain food festival

The Bahrain Food Festival is a gastronomic celebration coordinated annually by the Bahrain Tourism and Exhibition Authority, whose first edition was organised in 2016 [15]. The last edition of the festival was in 2022. It consists of more than 100 local and international foods that satisfy the tastes of local and international visitors celebration aims The celebration aims to promote Bahrain, as a destination for travellers for its culinary variety and provide guests with an impressive experience and spectacle (Bahrain New Agency BNA) [16]. The festival promotes the travel industry, particularly the field of conviviality and economy in Bahrain. Furthermore, this festival can contribute to achieving the goals of the Bahrain Travel Industry System 2022–2026. More than 200 activities accompanied the displays and festivities of the Bahrain Food Festival. These activities include a live cooking station with the participation of culinary pioneers (chefs from Bahrain) and a children’s play area where they can relax and have fun, musical and artistic performances with talented Bahraini singers. It also has a raffle for prizes in the completion area with the participation of Bahraini hotels and restaurants. Admission to the festival is free, allowing interested tourists to visit the site and enjoy the environment [13].

According to data from the Bahrain Tourism and Exhibition Authority [48], the Bahrain Food Festival in 2022 attracted 175,234 people for two weeks. The festival’s first week attracted over 75 thousand visitors, while the second week attracted more than 100 thousand. In addition, some guests returned to the festival several times to try various foods, sweets, and drinks prepared by 120 restaurants participating in the event [48]. According to statistics from the Bahrain Tourism and Exhibition Authority, the number of participants in the 2022 festival increased by 32% from the previous year due to large restaurant chains and independent restaurants run by entrepreneurs who chose hospitality as a career [48].

Bahrain has several traditional cuisines, such as Halwa, meaning sweets, and it has been backed for more than 200 years, according to Bahrain National Museum [49]. Halwa is like jelly, consisting of sugar, starch, oil, saffron, nuts, rose water, and cardamom. It i accompanied by Arabic coffee during family and friends’ gatherings and weddings. Another cuisine is Machboos, a dish of rice made with chicken, fish, or lamb with various, spices and dried lime. This dish entails cooking the rice and meat together, adding species, dried lime, rose water, and saffron [50]. Finally, the Bahraini Kebab is a vegetarian dish made of chickpea flour, spices, eggs, green chopped tomatoes, dill, and onions e shaped into tiny buns and fried in deep oil. The Bahrain Kebab is usually served throughout the year, but most are eaten during Ramadan [50].

## 4. Methodology

The present study had the following objectives for the Bahrain Food Festival: (i) Identify the motivational dimensions of the demand for the food festival, (ii) Determine the segments of the demand for the food festival, and (iii) Establish the relationship between the segments of demand and sociodemographic aspects. A questionnaire was designed to achieve these objectives. It consisted of socio-demographic aspects and motivations for attending the gastronomic festival. The first part included ten closed questions about the socio-demographic attributes of visitors obtained from the study by Lee et al. [43]. The second section analyzed the attendees’ motivations through 14 items obtained from Chang [27] and Carvache-Franco et al. [33]. These scales were developed using a 5-point Likert scale, with 1 equivalent to very little and 5 to a lot. Informed consent was requested in writing as part of the questionnaire. The study had ethical approval from ESPOL University.

A Google Forms survey was written in English and Arabic for online completion. For accessing the target population, the survey was developed in English first and translated, with back translation conducted by three bilingual scholars in the tourism and hospitality field. Then, a pre-test with 25 respondents was carried out. Based on the pilot study, improvement procedures were taken into consideration regarding the clarity of the survey instruction, the survey validity and whether it tests what it is intended to test, and finally check the timing for finishing the survey to ensure a better response of the survey. For instance, the personal information section in the survey was changed from fill-in-the-blank to closed-ended questions so that respondents can complete the survey in a reasonable amount of time and can be analyzed in the SPSS Program.

The survey was conducted via WhatsApp in Bahrain, a state in the Persian Gulf with a sizeable Muslim population and numerous ex-pats. This survey researched the motivations of visiting the festival, segmentation of attendees based on motivations, and segmentation relationship with socio-demographic variables (age, education, occupation, the accompany to the festival, monthly income,e and average expenditure per person in this visit). A convenience sampling methodology was applied for collecting data. Respondents were asked to leave their names and mobile number in a form made for this study. Then, the survey link was sent to the respondents. Forethical issues, it was confirmed to them that all data would be kept confidential and used only for research purposes.

The information was collected between March 19 and April 1, 2022. This study sample consisted of diverse individuals who indicated they had visited food festivals. In the current study, the targeted population consisted of locals and residents who came to Bahrain for work and non-residents who visit the festival to try the local and international dishes and to enjoy the festival activities, programme, and atmosphere. The festival was visited by more than 220,000 visitors in 2019. The researchers applied the finite population equation taking festival attendees in 2019 as the Universe, using a 5% margin of error, a certainty level of 95%, and a variance of 50%. Therefore, 380 valid questionnaires were obtained. The factor analysis, with a small number of factors, allowed the interpretation of the data and understanding the items. As an extraction technique, the main components method was used. The Varimax rotation method was used to organize the factor loadings.

Furthermore, the proposed model (consisting of motivations for visiting the festival, segmentation of visitors, and their sociodemographic characteristics) was investigated using the KMO (Kaiser-Meyer-Olkin) index and Bartlett’s sphericity test. The k-means clustering method was used in the second stage to identify relatively homogeneous case segments based on selected characteristics. Finally, the researchers used a Chi-square analysis to find the relationships between the segments and the socio-demographic variables. The information was coordinated, classified, and broken down with the SPSS variant 26.

## 5. Results

### 5.1. Respondent profile

This study’s sociodemographic variables show that national respondents (47.4%) and foreigners (52.6%) had similar attendance at the gastronomic festival. Moreover, a higher percentage of attendees were ale (59.2%). Most attendees were married (54.5%) and between 31 and 50 (49.7%). Likewise, most visitors had university qualifications (61.3%) and were private employees (47.4%). Also, 20.5% of attendees visited the gastronomic festival twice, and the other 20.5% visited more than three times. Most attendees (58.7%) visited the festival with family. Table 1 describes the data.

**Table 1:** Socio-demographic variables.

Variable		N	%
Nationality	National	180	47.4
	Foreign	200	52.6
Gender	Male	225	59.2
	Female	155	40.8
Marital Status	Single	107	28.2
	Married	207	54.5
	Others	66	17.4
Age	Less than 20 years	39	10.3
	21–30	103	27.1
	31–40	86	22.6
	41–50	103	27.1
	51–60	33	8.7
	More than 61 years	16	4.2
Education	Elementary	4	1.1
	High School	125	32.9
	Undergraduate	233	61.3
	Postgraduate/Maste/PhD	18	4.7
Occupation	Student	42	11.1
	Researcher/Scientist	2	0.5
	Businessman	16	4.2
	Private Employee	180	47.4
	Public Employee	49	12.9
	Pensioner	34	8.9
	Unemployed	51	13.4
	Other	6	1.6
How many times have you visited this festival?	First time	202	53.2
	Two times	78	20.5
	Three times	22	5.8
	More than three times	78	20.5
Who accompanied you?	Alone	44	11.6
	Family	223	58.7
	Friends	96	25.3
	Partner	15	3.9
	Other	2	0.5
What is your monthly income (dollars/month)?	Less than $500	103	27.1
	From $501 to $1000	16	4.2
	From $1001 to $1500	7	1.8
	From $1501 to $2000	8	2.1
	From $2001 to $2500	51	13.4
	From $2501 to $3000	88	23.2
	More than $3000	107	28.2
What was your average daily expenditure per person on this visit?	Less than $20	101	26.6
	From $20 to $40	25	6.6
	From $41 to $60	28	7.4
	From $61 to $80	85	22.4
	From $81 to $100	109	28.7
	More than $100	32	8.4

### 5.2. Motivations to attend the food festival

An exploratory factor analysis was performed to reduce the motivational items in a few factors allowing a more straightforward interpretation of the results. The data extraction technique was the principal component analysis. The Varimax rotation method was implemented to obtain an order of the factors, and the number of factors used in the Kaiser criteria included eigenvalues superior to 1. Five factors formed the art of the solution and explained 71.9% of the total variance. Cronbach’s alpha of the factors ranged from 0.844 to 0.536, indicating a high internal consistency in each factor. Factorial loads ranged from 0.544 to 0.898, so all factorial loads exceeded the critical value of 0.50 suggested by Hair et al. [51]. The Kaiser-Meyer-Olkin index (KMO) was 0.672, making it an acceptable value for the model. Likewise, Bartlett’s sphericity test was significant <0.05, so it was appropriate to apply factor analysis. Table 2 shows the results.

**Table 2:** Motivations in food festivals (Factor analysis).

Variable	Local Foods	Art	Entertainment	Socialization	Escape and novelty
To taste local food	0.883				
To get acquainted with local foods	0.858				
To exchange ideas about local foods	0.800				
To increase my knowledge about local foods	0.786				
To see the art show		0.898			
To see artists and musicians		0.887			
To buy handicrafts		0.544			
To have fun			0.850		
To enjoy the festive atmosphere			0.815		
To see and do a variety of things			0.620		
To observe other attendees				0.867	
To meet people with the same interests				0.857	
To have a change from my daily routine					0.819
To explore new restaurants					0.765
Cronbach’s alpha	0.844	0.707	0.653	0.712	0.536
Eigenvalue	3.207	2.516	1.900	1.298	1.146
Variance explained (%)	22.905	17.970	13.569	9.270	8.185
Cumulative variance explained (%)	22.905	40.875	54.444	63.714	71.899

According to Table 2, the first dimension (factor) was called Local food and was related to tasting local food, exchanging ideas, and increasing knowledge about this type of food. This dimension included 22.91% of the variance explained. Moreover, Art was the second dimension related to observing art shows with artists and musicians. This dimension corresponded to 17.97% of the variance explained. The third dimension was Entertainment which was related to the fun and enjoyment of the festive atmosphere. This dimension had 13.57% of the variance explained. Socialization was the fourth dimension because it relates to observing and meeting people who attend the gastronomic festival. This dimension included 9.27% of the total variance. The fifth dimension, Escape and novelty, relates to the novelty of knowing new restaurants and escaping from the daily routine. This dimension had 8.19% of the variance explained. The described results answer our first research question, RQ1: What are the motivations behind the visitor demand at the Bahrein food festival?

### 5.3. Segmentation in the gastronomic festival

An analysis of non-hierarchical K-medias grouping was used to gauge the demand at the food festival. This method helped maximize the variance between typologies and minimize the variance within each segment shown in Table 3.

**Table 3:** Segmentation in food festivals (K-medias Method).

Variable	Cluster 1: Escape and Novelty	Cluster 2: Multiple motives
To taste local food	3.2	4.9
To get acquainted with local foods	2.6	4.7
To explore new restaurants	4.9	4.9
To exchange ideas about local foods	4.0	4.9
To have a change from my daily routine	4.9	4.9
To see and do a variety of things	5.0	5.0
To enjoy the festive atmosphere	4.8	4.9
To have fun	4.8	5.0
To meet people with the same interests	4.1	3.9
To observe the other people attending the festival	2.7	2.5
To see the art show	3.8	4.5
To buy handicrafts	2.2	3.5
To see artists and musicians	3.3	4.3
To increase my knowledge about local foods	4.4	5.0

According to Table 3, the first segment, Escape, and novelty scored high in the motivations related to novelty and escape, such as enjoying the festive atmosphere, having fun, and knowing new restaurants. In contrast, the second segment scored high on all motivational items. It was called Multiple motives because it was a segment motivated by the gastronomic festival’s escape and novelty, the art, socialization, and local food visitors could find at the festival. The results answer our second research question, RQ2: What are the segments of visitor demand in the Bahrein food festival?

### 5.4. Demand segmentation and its relationship with socio-demographic variables

Pearson’s Chi-square test was used to find the significant relationships (p<0.05) between segments and socio-demographic variables. Table 4 presents the results.

**Table 4:** Segmentation and socio-demographic variables.

Variables		Cluster 1: Escape and Novelty	Cluster 2: Multiple motives	Total	Chi-square test
Age	Less than 20 years	17.1%	7.3%	10.3%	30,828; p<0.05
	21–30	23.1%	28.6%	26.9%	
	31–40	12.8%	27.1%	22.7%	
	41–50	29.1%	26.3%	27.2%	
	51–60	7.7%	9.2%	8.7%	
	More than 61 years	10.3%	1.5%	4.2%	
Education	Elementary	1.7%	0.8%	1.1%	44,946; p<0.05
	High School	56.4%	22.1%	32.7%	
	Undergraduate	39.3%	71.4%	61.5%	
	Postgraduate/Maste/PhD	2.6%	5.7%	4.7%	
Occupation	Student	17.9%	8.0%	11.1%	66,659; p<0.05
	Researcher/Scientist	0.9%	0.4%	0.5%	
	Businessman	4.3%	4.2%	4.2%	
	Private Employee	17.9%	60.7%	47.5%	
	Public Employee	25.6%	7.3%	12.9%	
	Pensioner	12.8%	7.3%	9.0%	
	Unemployed	18.8%	10.7%	13.2%	
	Other	1.7%	1.5%	1.6%	
Who accompanies you?	Alone	7.7%	13.4%	11.6%	14,113; p<0.05
	With your family	59.0%	58.4%	58.6%	
	With friends	33.3%	21.8%	25.3%	
	With your partner		5.7%	4.0%	
	Other		0.8%	0.5%	
What is your monthly income (dollars/month)?	Less than USD 500	38.5%	21.8%	26.9%	15,182; p<0.05
	From $501 to $1000	2.6%	5.0%	4.2%	
	From $1001 to $1500	1.7%	1.9%	1.8%	
	From $1501 to $2000	1.7%	2.3%	2.1%	
	From $2001 to $2500	14.5%	13.0%	13.5%	
	From $2501 to $3000	22.2%	23.7%	23.2%	
	More than $3000	18.8%	32.4%	28.2%	
What was your average daily expenditure per person on this visit?	Less than $20	37.6%	21.4%	26.4%	
	From $20 to $40	6.0%	6.9%	6.6%	12,318; p<0.05
	From $41 to $60	7.7%	7.3%	7.4%	
	From $61 to $80	18.8%	24.0%	22.4%	
	From $81 to $100	24.8%	30.5%	28.8%	
	More than $100	5.1%	9.9%	8.4%	

According to Table 4, the Escape and novelty segment had a higher percentage (17.1%) of food festival attendees under 20 compared to the other segment. Likewise, this segment included a higher percentage of attendees (10.3%) over 61 years of age than the other group. In contrast, the Multiple motives segment had a higher percentage of attendees to the gastronomic festival from 31 to 40 years old than the other segment. Concerning education, the segment Escape and novelty included 56.74% of festival attendees with a high school level education. In comparison, the segment Multiple Motives had 71.4% of attendees who held undergraduate qualifications, being the segment with the highest level of education. According to occupation, the Escape and novelty segment had 25% of public employees, while 18% of attendees were unemployed. Regarding the company with whom they traveled to the festival, the segment Escape and novelty primarily traveled with their family and friends. In contrast, the segment with Multiple motives traveled with their family and friends, alone or with their partner. Hence, it was the segment with the greatest companion type diversity. According to the monthly income, the Escape and novelty segment had the highest percentage of attendees (38.5%) with revenues of less than $500. In comparison, the Multiple motives segment included the highest percentage (32.4%) of attendees who received a monthly income greater than $3000, making it the segment with the highest monthly income. Regarding the average daily expenditure per person, the Escape and novelty segment had the highest percentage of attendees to the gastronomic festival (37.6%), with expenses of less than $20. In comparison, the Multiple motives segment included a higher percentage of attendees (30.5%) who received monthly income between $81 and $100, so it was the segment that spent the most on the festival. The results that answer our third research question, RQ3: What is the relationship between the visitor demand segments and their sociodemographic variables in the Bahrein food festival?

## 6. Discussion

The first objective of this study was to identify the motivational dimensions of the demand for the Bahrain food festival. The results show five primary motivational dimensions to visit the Bahrain food festival: Local food, art, entertainment, socializing, and escape and novelty in response to RQ1. This outcome coincides with previous studies that established various motivations for visiting food festivals, such as enjoying the event, tasting food, spending time with family, and escaping daily routine. Moreover, those motivations included: enjoying a new dining experience (tasting food), culture (learning about a different culture and tasting local food), and socialization (increasing family and friendship ties and exchanging ideas about food with other visitors) [22, 24, 33]. Likewise, Carvache-Franco et al. [33] found that motivations for visiting the Red Crab festival in Ecuador included escape, socialization, art, food, local food, and knowledge.

Similarly, Kabiraj et al. [37] pointed out two motivations for attending the Dalian International Wine and Dining Festival. Those sets are Primary Motivations like tasting and buying wine and food, experiencing wine, learning food culture, and attending specific activities like wine and food tasting. Secondary motivations include being with family, meeting new friends with the same interests, and releasing pressures and changes in daily routine. Also, Chang [27] found similar motivations, such as escape/novel event and socializing, in the present study. Also, academics Hermann et al. [40] found similar motivations, such as gastronomy, entertainment, and socialization at a food festival. The contribution of this study to the academic literature on food festivals consists in identifying that the motivations in gastronomic tourism are multidimensional, and tourists’ visit is determined by different factors that vary from one festival to another. More importantly, it contributed to understanding the different reasons for visiting the food festival in Bahrain, as this is the first study conducted in Bahrain and the Arabian Gulf region. This study may assist in understanding why the motivations to visit food festivals vary because of differences in the themes and programs that make specific tourists visit specific food festivals such as the Bahrain Food Festival.

The study’s second objective was to determine the demand segments for the Bahrain food festival. In response to RQ2, the results show two segments based on attendees’ motivations. The first is the Escape and novelty segment for attendees looking to enjoy the festive atmosphere and discover new restaurants. In contrast, the Multiple Motives segment is made up of attendees who have several motivations at the same time. This outcome contrasts with the available studies on this relationship. For example, Sohn and Yuan [44] identified five groups of visitors to the Lubbock Wine Festival in Texas: Idealists, Achievers, Explorers, Belongers, and Innovators. Along the same lines, Carvache-Franco et al. [33] identified three segments of attendees at the Red Crab Festival in Ecuador. These are escape and food seekers, passive visitors, and multiple motives. Similarly, Viljoen et al. [41] found a group of "Social Epicureans" attracted to escape and socialization. Similar to our segment exhaust and novelty. A second group, the "Serious epicureans, were motivated by a complete experience similar to our group of multiple motives, and the "selective epicureans" were not found in our study.

The present study’s contribution to the academic literature is finding new segments of demand unrevealed in previous research. Previous studies have found multiple motivations. However, in this study, each demand segment is based on more standardized motivations. The first segment is based on the motivation to enjoy the atmosphere, while the second segment, Multiple Motives, is based on various motivations.

The last objective of this research was to identify the relationship between demand segmentation and socio-demographic aspects. This study has found a significant difference between the two segments of visitors to the Bahrain food festival. In terms of age, the Entertainment segment has the youngest and oldest age of visitors compared to the Multiple motives segment. Regarding education, Multiples motives attendees had college and higher education and the highest monthly income. Therefore, these attendees spent the most during the festival.

A careful look at the literature in this manuscript on the relationship between socio-demographic aspects and the segmentation of the demands of food festival attendees showed different results from this study. This research contributes to the outcome that shows how the socio-demographic characteristics of festival goers vary between two demand segments. Previous studies identified multiple demand segments with various socio-demographic characteristics factors. Additionally, this study contributed by basing those segments on motivations and socio-demographics rather than on satisfaction and experiential value, as in previous studies.

In addition, the study has practical implications. First, the results show how the food festival has become a significant tourist attraction in Bahrain, a growing tourist destination. From this point of view, this work’s main contribution is to better understand the differentiating characteristics of the visitor segments, their motivations on the one hand and their sociodemographic factors on the other, in food festivals. This outcome allows the organizers to plan better, organize, and promote the festival in terms of program, services, facilities, and products to adapt to the segmentation of demands, sociodemographic characteristics, and visitors’ motivations. This study encourages the organizers of the Bahrain food festival to create an annual theme for the festival, such as local food, to attract more interested visitors. Practically, this study can provide marketers and tourism industry workers, such as travel agencies and tourist activities organisers, and, the opportunity to promote Bahrain’s traditional food. Therefore, more promotions can be arranged in collaboration with prestigious Bahraini traditional restaurants and famous food bloggers to ensure the engagement of more visitors not only to the festival but to Bahrain in general. Finally, Organisers of the festival can add more cultural activities and events into the festival, not just musical concerts but also classic old games and a traditional fashion show to engage and satisfy those who visit Bahrain to learn entertain about its culture and those visiting for the first time.

Furthermore, it is essential to understand the motivations and expectations of visitors to the Bahrain Food Festival. Festival organizers should address local food, art, and entertainment to promote fun and enjoyment for visitors. The Bahrain Food Festival organizers promote these visitors’ many motives by regularly researching their needs and planning services to meet their different needs. Bahrain Food Festival organizers and planners can meet the demands of different visitor segments by improving services, facilities, and event themes to encourage more visitors.

## 7. Conclusions

Food festivals have been a growing tourism sector in these years due to their contributions to the region’s economic, marketing, brand, and social growth. They also raise awareness of the place among tourists, improve their perception of the destination and provide information about the local culture. Studying the visitors’ motivations for these festivals is essential before planning the process to provide an opportunity for creating programs that motivate festivalgoers. At the same time, it is vital to classify festival attendees according to their characteristics and interests in segments to ensure the provision of services, programs, and schedules appropriate to their needs.

Among the results of this study, we found that the motivations in the Bahrain food festival consist of five dimensions: Local food, Art, Entertainment, Socialization, and Escape and novelty of the event. In contrast, the demand for the Bahrain food festival comprises two segments. The first is the Entertainment segment related to attendees looking to enjoy the festive atmosphere and discover new restaurants. The second segment is the multi-motive segment, which is made up of attendees who have several motivations at the same time. This segment has the highest income and expenses in food festivals, so it is the most critical segment for elaborating plans and strategies.

Theoretically, the results mark a significant advance in food festival research. Previous research has mainly focused on the general motivation of visitors to food festivals, with little importance given to the segmentation of demands, which represent a large area and growth in tourism companies. This study provides evidence of the effect of experience by showing how socio-demographic characteristics of respondents, such as age, education, marital status, job, and average daily spending per person, affect demand segmentation. This study found that socio-demographic factors significantly impacted visitor motivations and demand segmentation, particularly age and average daily expenditure per person. The influence of socio-demographic characteristics varies from one demand segment to another, depending on the motivation of each one.

More importantly, this study is one of the few that investigates in a single analysis a construct formed by the motivations, the segmentation of the demands, and the sociodemographic aspects of the visitors to the gastronomic festival. For this reason, studies on these variables are scarce. Therefore, this study opens new doors for similar studies in the Arab World and the Gulf Region and facilitates the understanding and nature of those festivals and visitors. The findings add significant knowledge to the academic literature on consumer behavior, food festivals, motivation, and demand segmentation. This study contributes to science because it is the first to focus on the Bahrain food festival regarding its motivations, demand segmentation, and socio-demographic aspects. The present study also identified different sets of demand segmentations from other studies based on visitor motivations: escape and novelty, including those visitors looking to enjoy the festive atmosphere and discover new restaurants. In contrast, the Multiple Motives segment includes visitors with multiple motivations simultaneously. In addition, this study found that the socio-demographic characteristics of festival attendees varied only between two demand segments.

This study has some limitations. This research focuses on the Bahrain food festival. Other food festivals in the Gulf region, such as the Dubai Food Festival and the Doha Food Festival, can be researched and compared because each country has its economic capabilities that can affect the organization and planning of the festival. This research studied the segmentation of demands based on motivations. There are other bases to explorethe segmentation of demands, such as lifestyle and satisfaction. New studies could be performed based on other factors different from reasons to bring new knowledge to this research area. This study was limited to festival visitors and did not include exhibitors such as chefs, musicians, and artists. The study also used the Convenience method by arbitrarily choosing the participants within the study. As a future line of research, it will be beneficial to study the socio-demographic profile of the exhibitors with the motivations for participating in the event and the segmentation of demands. Thus, the festival’s planning and organization will provide suitable venues, services, financial support, and visitor sponsorship.

## Supporting information

S1 File (SAV)

## References

[1] Chatzinakos G. Exploring potentials for culinary tourism through a food festival: The case of Thessaloniki Food Festival. Transnational Mark. J. (TMJ). 2016; 4(2), 110–125. doi: 10.33182/tmj.v4i2.394

[2] Fontefrancesco MF. Festive Gastronomy Against Rural Disruption: Food Festivals as a Gastronomic Strategy Against Social-Cultural Marginalization in Northern Italy. 2020; 25(1), 3–17. 10.1080/15528014.2021.1884421

[3] Fontefrancesco MF. Food festivals in Italy: a festive strategy against rural marginalization. Food Cul. Soc. 2022; 25(1), 3–17. doi: 10.1080/15528014.2021.1884421

[4] Fontefrancesco MF, Zocchi DM. Reviving traditional food knowledge through food Festivals. The case of the pink asparagus Festival in Mezzago, Italy. Front Sustain Food Syst. 2020; 4,1–10. 10.3389/fsufs.2020.596028

[5] Yang FX, Wong IA, Tan XS, Wu DCW. The role of food festivals in branding culinary destinations. Tour. Manag. Perspect. 2020; 34, 100671. 10.1016/j.tmp.2020.100671

[6] Akgunduz Y, Coşar Y. Motivations of event tourism participants and behavioural intentions. Tour. Hosp Manag. 2018; 24(2), 341–358. 10.20867/thm.24.2.4

[7] Pérez-Gálvez JC, Medina-Viruel MJ, C Jara-Alba, López-Guzmán T. Segmentation of food market visitors in World Heritage Sites. Case study of the city of Córdoba (Spain). Curr Issues Tour. 2021; 24(8), 1139–1153. 10.1080/13683500.2020.1769570

[8] Hsu FC, Park SH, Miller JC. Segmenting food festivalgoers: experiential value, emotional state and loyalty. Bri. Food J. 2022; 125(1), 29–48. 10.1108/BFJ-05-2021-0549

[9] Kim YG, Eves A. Scarles C. Building a model of local food consumption on trips and holidays: a grounded theory approach. Int. J. Hosp. Manag. 2009; 28(3), 423–431. 10.1016/j.ijhm.2008.11.005

[10] Bašan, L, Lončarić D, Čuić Tanković, A. Local residents as visitors to exhibitions: investigating motivation and attitudes towards traditional food festivals. In Recent Advances in Applied Economics, Proceedings of the 6th International Conference on Applied Economics and Development (AEBD’14), 2014, pp. 27–33. At: Lisabon, Portugal, 30.10.-1.11.2014

[11] Robinson RN, Getz D. Profiling potential food tourists: An Australian study. Bri. Food J. 2014; 116(4), 690–706. 10.1108/BFJ-02-2012-0030

[12] Chanda RS, Pabalkar V, Gandhi P. Effect of consumer’s demographics and attitude on food festival. Indian J. Ecol. 2020; 47(spl), 42–46. Available at https://www.indianjournals.com/ijor.aspx?target=ijor:ije1&volume=47&issue=spl&article=007

[13] Calendar. 2022. Available at https://calendar.bh/en/bahrain-food-festival-2-3-4-5-6-7

[14] Bizbahrain, sf. Available at https://www.bizbahrain.com/73,000-visitors-attends-Food-Festival-2022-in-the-first-week-bizbahrain

[15] Bizbahrain, sf. Available at https://www.bizbahrain.com//bahrain-food-festival-kicks-off/

[16] Bna. sf. Available at https://www.bna.bh/en/news?cms=q8FmFJgiscL2fwIzON1%2BDpk3DzETxFgsr%2FmQUX95MsI%3D

[17] Kim YH, Duncan JL, Jai TM. A case study of a southern food festival: using a cluster analysis approach. Anatolia. 2014; 25(3), 457–473. 10.1080/13032917.2014.912245

[18] Organ K, Koenig-Lewis N, Palmer A, Probert J. Festivals as agents for behaviour change: A study of food festival engagement and subsequent food choices. Tour. Manag. 2015; 48, 84–99. 10.1016/j.tourman.2014.10.021

[19] Björk P, Kauppinen-Räisänen H. Culinary-gastronomic tourism—a search for local food experiences. Nut. Food Sci. 2014; 44(4), 294–309. 10.1108/NFS-12-2013-0142

[20] Duran E, Hamarat B. Festival attendees’ motivations: the case of International Troia Festival. Int. J. Event Festiv. Manag. 2014; 5(2), 146–163. 10.1108/IJEFM-07-2012-0020

[21] Moutinho L. Consumer Behaviour, in: Moutinho L. (ed.), Strategic Management in Tourism, CABI Publishing, New York. 2000

[22] López-Guzmán T, Lotero CPU, Gálvez JCP, Rivera IR. Food festivals: Attitude, motivation and satisfaction of the tourist. Bri Food J. 2017; 119 (2), 267–283. 10.1108/BFJ-06-2016-0246

[23] Krajíčková A, Šauer M. Differences in motivation of food festivals visitors–A view from the Czech Republic. Geogr. Pannonica. 2018; 22(3). 10.5937/gp22-17050

[24] Horng JS, Su CS, So SIA. Segmenting food festival visitors: applying the theory of planned behaviour and lifestyle. J. Conv. Event Tour. 2013; 14 (3), 193–216. 10.1080/15470148.2013.814038

[25] Kim S, Savinovic A, Brown S. Visitors’ Motivations in Attending an Ethnic Minority Cultural Festival: A Case Study of the Feŝta Croatian Food and Wine Festival, South Australia. Event Manag. 2013; 17(4), 349–359. 10.3727/152599513X13769392444828

[26] Hattingh C, Swart K. The motives for visitors to attend a food and wine event in Cape Town and their satisfaction levels. African J. Hosp. Tour. Leis. 2016; 5(2), 1–14. http://hd1.handle.net/11189/7547

[27] Chang W. A taste of tourism: Visitors’ motivations to attend a food festival. Event Manag. 2011; 15(2), 151–161. 10.3727/152599511X13082349958190

[28] Kim S, Lee C. Push and pull relationships. Ann Tour. Res. 2002; 29(1), 257–260. 10.1016/S0160-7383(01)00043-3

[29] Yuan JJ, Cai LA, Morrison AM, Linton S. An analysis of wine festival attendees’ motivations: A synergy of wine, travel and special events. J. Vacat Mark. 2005; 11(1), 41–58. 10.1177/1356766705050842

[30] Smith S, Costello C, Muenchen RA. Influence of push and pull motivations on satisfaction and behavioral intentions within a culinary tourism event. J. Quality Assu Hosp. Tour. 2010; 11(1), 17–35. 10.1080/15280080903520584

[31] Park KS, Reisinger Y, Kang HJ. Visitors’ motivation for attending the South Beach wine and food festival, Miami Beach, Florida. J. Travel Tour. Mark. 2008; 25(2), 161–181. 10.1080/10548400802402883

[32] Topole M, Pipan P, Gašperič P, Geršič M, Kumer P. Culinary events in the Slovenian countryside: Visitors’ motives, satisfaction, and views on sustainability. Acta Geographica Slovenica. 2021; 61(1), 107–125. https://orcid.org/0000-0003-1007-2289

[33] Carvache-Franco M, Carvache-Franco O, Carvache-Franco W, Villagómez-Buele C, Arteaga-Peñafiel M. Motivation and segmentation of gastronomic events: festival of the Red Crab in Ecuador. Ann Leis. Res. 2020; 1–17. 10.1080/11745398.2020.1830421

[34] Hu Y, Banyai M, Smith S. Expenditures, Motivations, and Food Involvement Among Food Festival Visitors: The Hefei, China, Crawfish Festival: 美食节访客的消费力, 参与推动力及食物品尝情况: 中国合肥的小龙虾节. J. China Tour. Res. 2013; 9(4), 467–488. 10.1080/19388160.2013.839410

[35] O’Regan M, Choe J, Yap M. Attendee motivations at an international wine festival in China. Event Manag. 2017; 21(4), 448–460. 10.3727/152599517X14998876105774

[36] İrigüler F, Özdoğan ON. Food festivals as a gastronomic event and attendees’motivations: The case of international urla artichoke festival. Int. J. Contemp Tour. Res. 2017; 5(1), 77–86. 10.30625/ijctr.834689

[37] Kabiraj S, Upadhya A, Vij A. Exploring the factors affecting the behavioral intention of visitors in wine festival: the case of China Dalian international wine and dine festival. Bus. Perspect Res. 2021; 9(3), 352–369. https://doi.org/10.1177%2F2278533721989521

[38] Lee W, Kwon H. The Influence of Personal Involvement on Festival Attendees’ Revisit Intention: Food and Wine Attendees’ Perspective. Sustainability. 2021; 13(14), 7727. 10.3390/su13147727

[39] Castillo-Canalejo AM, Sánchez-Cañizares SM, Santos-Roldán L, Muñoz-Fernández GA. Food markets: A motivation-based segmentation of tourists. Int. J. Environ Res. Public Health. 2020; 17(7), 2312. doi: 10.3390/ijerph17072312 32235438 PMC7178125

[40] Hermann UP, Boshoff L, Ncala TT. Understanding beer festival attendee motivations to a craft beer festival in South Africa. J. New Generation Sci. 2019; 17(2), 18–30. https://hdl.handle.net/10520/EJC-1cb1e33f06

[41] Viljoen A, Kruger M, Saayman M. The 3-S typology of South African culinary festival visitors. Int. J. Contemp. Hosp. Manag. 2017; 29 (6), 1560–1579. 10.1108/IJCHM-09-2015-0464

[42] Björk P, Kauppinen-Räisänen H. Exploring the multi-dimensionality of travellers’ culinary-gastronomic experiences. Curr. Issues Tour. 2016; 19(12), 1260–1280. 10.1080/13683500.2013.868412

[43] Lee S, Lee S, Lee G. Ecotourists’ motivation and revisit intention: a case study of restored ecological parks in South Korea. Asia Pacific J. Tour. Res. 2014; 19(11), 1327–1344. 10.1080/10941665.2013.852117

[44] Sohn E, Yuan JJ. Who are the culinary tourists? An observation at a food and wine festival. Int. J. Cul. Tour. Hosp Res. 2013. 10.1108/IJCTHR-04-2013-0019

[45] De Albuquerque Meneguel CR, Mundet L, Aulet S. The role of a high-quality restaurant in stimulating the creation and development of gastronomy tourism. Int. J. Hosp Manag. 2019; 83, 220–228. 10.1016/j.ijhm.2018.10.018

[46] Kim YG, Eves A Scarles C. Empirical verification of a conceptual model of local consumption at a tourist destination. Int. J. Hosp. Manag. 2013; 33, 484–489. 10.1016/j.ijhm.2012.06.005

[47] Saayman M, Saayman A, Joubert EM. Expenditure-based segmentation of visitors to the Wacky Wine Festival. Tour. Recreat. Res. 2015; 37(3), 215–225. 10.1080/02508281.2012.11081710

[48] Bahrain Tourism and Exhibition Authority. 2022. Available at http://www.btea.bh/news-details?newsld=56715

[49] Culture. Sf. Available at https://www.culture.gov.bh/en/events/AnnualFestivalsandEvents/HeritageFestival/HeritageFestival2016/Food/

[50] Travel Food Datlas. sf. Available at https://travelfoodatlas.com/bahrain-food

[51] Hair JF, Ortinau DJ, Harrison DE. Essentials of marketing research (Vol. 2). New York, NY: McGraw-Hill/Irwin. 2010

